# Molten Salt Synthesis of High-Performance, Nanostructured La_0.6_Sr_0.4_FeO_3−δ_ Oxygen Electrode of a Reversible Solid Oxide Cell

**DOI:** 10.3390/ma13102267

**Published:** 2020-05-14

**Authors:** Xiaodong Zuo, Zhiyi Chen, Chengzhi Guan, Kongfa Chen, Sanzhao Song, Guoping Xiao, Yuepeng Pang, Jian-Qiang Wang

**Affiliations:** 1 School of Materials Science and Engineering, University of Shanghai for Science and Technology, Shanghai 200093, China; zxd_usst@163.com; 2 Key Laboratory of Interfacial Physics and Technology, Shanghai Institute of Applied Physics, Chinese Academy of Sciences, Shanghai 201800, China; songsanzhao@sinap.ac.cn (S.S.); xiaoguoping@sinap.ac.cn (G.X.); 3 College of Materials Science and Engineering, Fuzhou University, Fuzhou, Fujian 350108, China; zhiyichen163@163.com; 4 Dalian National Laboratory for Clean Energy, Dalian 116023, China

**Keywords:** reversible solid oxide fuel cell, molten salt synthesis, interface, LSF oxygen electrode, direct assembly

## Abstract

Nanoscale perovskite oxides with enhanced electrocatalytic activities have been widely used as oxygen electrodes of reversible solid oxide cells (RSOC). Here, La_0.6_Sr_0.4_FeO_3−δ_ (LSF) nanoscale powder is synthesized via a novel molten salt method using chlorides as the reaction medium and fired at 850 °C for 5 h after removing the additives. A direct assembly method is employed to fabricate the LSF electrode without a pre-sintering process at high temperature. The microstructure characterization ensures that the direct assembly process will not damage the porosity of LSF. When operating as a solid oxide fuel cell (SOFC), the LSF cell exhibits a peak power density of 1.36, 1.07 and 0.7 W/cm^2^ at 800, 750 and 700 °C, respectively, while in solid oxide electrolysis cell (SOEC) mode, the electrolysis current density reaches 1.52, 0.98 and 0.53 A/cm^2^ under an electrolysis voltage of 1.3 V, respectively. Thus, it indicates that the molten salt routine is a promising method for the synthesis of highly active perovskite LSF powders for directly assembled oxygen electrodes of RSOC.

## 1. Introduction

Reversible solid oxide cells (RSOC) are a novel energy conversion and storage device with the highest efficiency [1]. In solid oxide fuel cell (SOFC) mode, they can directly convert the chemical energy of clean fuels into electricity and heat [2], while in solid oxide electrolysis cell (SOEC) mode, they can convert electrical and thermal energy into chemical energy for storage [3]. The oxygen electrode, where the oxygen reduction reaction (ORR) and oxygen evolution reaction (OER) occur, is one of the dominant components that affect the overall performance and stability of the RSOC [4]. As a mixed electronic and ionic conductor (MIEC), (La,Sr)FeO_3−δ_ (LSF) has been extensively studied to replace the conventional manganite based materials and exhibit much higher electrochemical performance at intermediate temperature (600–800 °C) [5,6]. LSF has a similar thermal expansion coefficient (TEC) to the zirconia-based electrolyte, as well as improved stability and lowered cost, compared to Sr-doped LaCoO_3_ material [7].

In general, decreasing the size of the electrode material grains will enlarge the specific surface and promote the electrochemical activities. For example, Fan et al. introduced nanosized La_0.6_Sr_0.4_FeO_3−δ_ particles into YSZ scaffold via the infiltration method to fabricate nanostructured LSF/YSZ oxygen electrodes after firing at 850 °C for 5 h. The infiltrated cell exhibits a peak power density of 611 mW/cm^2^ using humidified H_2_ at 800 °C [8]. Another effective technology to fabricate perovskite electrocatalysts is the molten salt synthesis (MSS) method. Xue et al. have summarized some progress in the synthesis, characterization and potential applications of low-dimensional perovskite oxide nanostructures by the MSS method [9]. Song et al. obtained porous iron-rich La_0.6_Sr_0.4_Co_0.2_Fe_0.8_O_2.9_ (LSCF) at a lowered temperature of 850 °C, with enriched surface area and high activity [10]. Guan et al. employed the MSS method to synthesize Nb-doped LSF electrode for RSOC, achieving a peak power density of 0.79W/cm^2^ in SOFC mode and an electrolysis current density of 0.89A/cm^2^ in SOEC mode at 800 °C [11]. However, if the nanometric powders are for use as the oxygen electrode of RSOC, the negative effect of the conventional pre-sintering at the temperature of 1000–1200 °C on microstructural coarsening is not negligible [12]. High-temperature sintering accelerates Sr surface segregation (SSS) [13], which is the cause of formation of insulating SrZrO_3_ phases at the interface between the electrode and electrolyte. The SSS also leads to formation of SrCrO_4_ layers on the surface of the electrode [14,15]. Therefore, to mitigate the SSS phenomenon in the oxygen electrode, reducing the presintering temperature is favorable. An alternative strategy to lower the fabrication temperature is to use the direct assembly technique by applying a polarization current [16,17,18]. Ai et al. manufactured the LSCF electrode on barrier-layer-free yttria-stabilized zirconia (YSZ) electrolyte without presintering, achieving a current density of 1.5 A/cm^2^ at 750 °C and 1.26 V in 50% H_2_O/50% H_2_ [19]. The advantages of the directly assembled electrode also include the preservation of particle-shaped contact in the interface and the nanostructure of the particles [16], sharing the same effect of the infiltration process.

In this study, we use the MSS method to synthesize nanoscale La_0.6_Sr_0.4_FeO_3−δ_ (LSF) material for the oxygen electrode of a directly assembled RSOC. Chloride salts are added in the reactants, forming a liquid environment to accelerate the reaction at a reduced temperature, and the added chlorides are removed by cleaning the resultant powders. The measurement results show that the directly assembled LSF electrode exhibits attractive electrochemical performance in both SOFC and SOEC modes.

## 2. Materials and Methods

### 2.1. Synthesis and Characterization of LSF Powders

**Figure 1:**
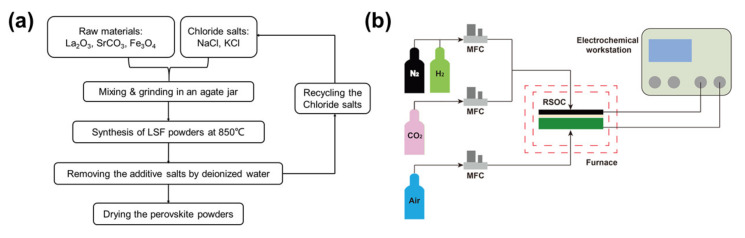
Schematic diagrams of molten salt synthesis procedures of La_0.6_Sr_0.4_FeO_3−δ_ (LSF) powders (**a**) and the button cell test apparatus (**b**).

The procedure of the MSS method used for the synthesis of LSF powders is shown in Figure 1a. La_2_O_3_ (99.9%, Aladdin, Shanghai, China), SrCO_3_ (99.9%, Alfa Aesar, Shanghai, China), Fe_3_O_4_ (99.9%, Adams Reagent Co Ltd., Shanghai, China), NaCl (99.9%, Aladdin) and KCl (99.9%, Aladdin) were used as the reactants and additives and mixed in an agate mortar by grinding. The weight ratio between the overall reactants and the salts was 1:5, while the molar ratio of NaCl and KCl was 1:1. The mixture was fired at 850 °C in air for 5 h. The chloride mixture can be liquidized at the firing temperature, leading to significantly enhanced motilities of the reactants, in contrast to the case of conventional solid-state reaction. After washing the resultant powder with deionized water using the centrifugal process, the obtained LSF powders were dried at 150 °C for 24 h, and the added salts could be recycled.

To confirm the phase evolution of as-synthesized and heat-treated LSF powders, X-ray diffraction (XRD) data were collected on a Bruker D8 Advance X-ray diffractometer (Karlsruhe, Baden-Württemberg, Germany) using the Ni-filtered Cu Kα radiation source at 40 kV and 40 mA (λ = 1.5148 Å), employing a scan rate of 1.5°/min in the 2θ range from 20 to 80°. Scanning electron microscopy (SEM) measurements were carried out using a Zeiss Merlin Compact LEO 1530 VP SEM (Jena, Thüringen, Germany). Transmission electron microscopy (TEM) characterization was carried out with FEI Tecnai G2 F20 electron microscopy (Hillsboro, OR, USA). X-ray photoelectron spectrometry (XPS) was utilized to evaluate the valence state of the elements in LSF with a Thermo Scientific ESCA-LAB 250Xi analyzer (Hillsboro, OR, USA), and the energy scale was internally calibrated by setting the C 1 s peak at 284.6 eV.

### 2.2. Fabrication and Measurement of the Button Cell with Directly Assembled LSF Electrode

A NiO (J.T. Baker, Avantor, Allentown, PA, USA)-YSZ (8 mol% Y_2_O_3_ stabilized zirconia, Tosoh, Shiba, Tokyo, Japan) hydrogen electrode-supported YSZ electrolyte film button cell was fabricated using slurry spin coating (VTC-100, MTI, Shenyang, Liaoning, China) and co-sintered at 1450 °C in air for 5 h. A Gd_0.1_Ce_0.9_O_1.95_ (GDC, AGC Seimi Chemical Co Ltd., Chigasaki, Kanagawa, Japan) interlayer was prepared on the YSZ film by slurry spin coating and sintered at 1250 °C in air for 2 h. LSF ink was prepared by mixing the porous LSF powders with an ink vehicle (VEH, Fuel Cell Materials) and screen-printed on the YSZ/GDC bilayer electrolyte films. The directly assembled LSF electrode was dried in an oven at 100 °C for 2 h without further pre-sintering process. The effective area of the oxygen electrode was 0.22 cm^2^.

The procedures of sealing and testing of the button cell in both SOFC mode and SOEC mode have been described in detail elsewhere [19]. Prior to the testing, the directly assembled LSF electrode was polarized at a constant current density of 0.5 A/cm^2^ and 750 °C under anodic mode to form the interface of the oxygen electrode and the electrolyte. Then, the measurement of the polarization performance and electrochemical impedance spectra (EIS) from 800 to 700 °C was carried out using a self-made testing system, composed of several gas mass flowmeters, a furnace and a Gamry Interface 1000E Potentiostat (Gamry Instruments, Warminster, PA, USA), as shown in Figure 1b. EIS data were collected in the frequency range of 100 k–0.01 Hz with an AC signal of 10 mV under open circuit conditions. The high-frequency intercept of EIS represents the ohmic resistance (R_Ω_) and the difference between low- and high-frequency intercepts is related to the electrode polarization resistance (R_p_).

## 3. Results

### 3.1. Characterization of LSF Powders

**Figure 2:**
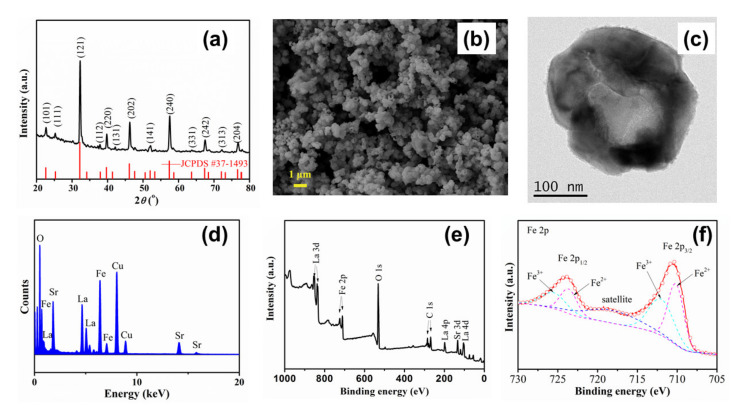
(**a**) XRD patterns of LSF powders; (**b**) SEM, (**c**) TEM images and (**d**) EDS spectrum of the particles; (**e**) overall XPS spectrum and (**f**) Fe 2p core level of LSF.

The phase purity of the as-synthesized LSF powders was characterized by room temperature XRD. The crystallographic structure of all the powders, shown in Figure 2a, can be indexed with the orthorhombic perovskite phase belonging to the Pnma (62) space group (JCPDS no.37–1493). The unit cell lattice parameters were refined and calculated to be a = 5.553, b = 7.845 and c = 5.561 Å. The XRD patterns indicate that all the additive chlorides can be recycled. However, an extra shoulder peak next to the (121) reflection of LSF is observed, and it can be attributed to trace La-Sr-Fe solid solution. Figure 2b,c gives the SEM and TEM morphologies of LSF particles and reveals their porous structure. The particle size of LSF powders without ball-milling is between 100 and 300 nm, with lots of holes in the grains to form an obvious nanobowl-shaped structure. Additionally, the diameter of the holes is about 100 nm. The origin of the holes could be complex, including the corrosion of metals in the molten salt, the removal of the salt during the washing process, or the evaporation of gases and salts. However, there is no doubt that the porosity of the particles will enlarge the specific area and the number of active sites [10]. The EDS spectrum (Figure 2d) of the powders also confirms that all the additive chloride salts have been removed, and the overall XPS spectrum (Figure 2e) reveals the presence of elements of La, Sr, Fe and O. The core level spectrum of Fe 2p is displayed in Figure 2f and splits into two parts, Fe 2p_3/2_ and Fe 2p_1/2_. The main Fe 2p_3/2_ peak occurs at 710.7 eV, while the Fe 2p_1/2_ peak is observed at 724.0 eV. Based on the fitting and analysis of Fe 2p core level, Fe exists in mixed valence states of Fe^2+^ and Fe^3+^, and the relative molar ratio of Fe^2+^/Fe^3+^ is 1.12 [20,21,22,23]. As is reported, the lowered valence state of the transition metal Fe determines better oxygen ionic conductivity and catalytic activity of LSF, since the existing Fe^2+^ would induce the formation of oxygen vacancies [24,25].

### 3.2. Electrochemical Performance Measurement

#### 3.2.1. SOFC Performance

**Figure 3:**
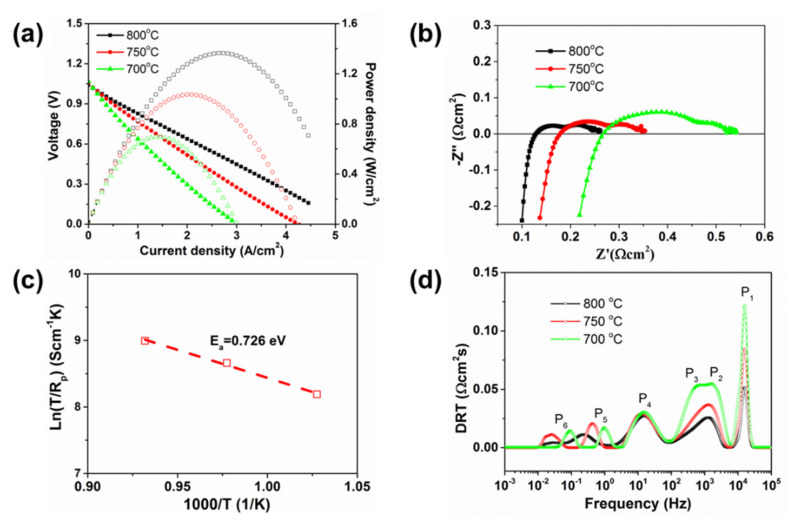
(**a**) I–V discharge curves, (**b**) the impedance spectra, (**c**) the activation energy for the polarization process and (**d**) the distribution of relaxation times (DRT) analysis of the impedance spectra of the directly assembled LSF cell in solid oxide fuel cell (SOFC) mode at 700–800 °C.

Figure 3a shows the current–voltage–power curves of the button cell with directly assembled LSF oxygen electrode in SOFC mode, using H_2_ and air as fuel and oxidant. The initial maximum power densities (MPDs) are 1.36, 1.03 and 0.7 W/cm^2^ at 800, 750 and 700 °C, respectively. It is highlighted that the performance of the directly assembled LSF cell is comparable to presintered cells with cobalt-free electrodes, such as infiltrated La_0.6_Sr_0.4_FeO_3−δ_ (611 mW/cm^2^ at 800 °C) and Nd_2_NiO_4_ (700 mW/cm^2^ at 700 °C), La_0.8_Sr_0.2_FeO_3−δ_ prepared using the glycine-nitrate combustion method (950 mW/cm^2^ at 750 °C), as well as La_0.6_Sr_0.4_Fe_0.9_Nb_0.1_O_3−δ_ (503 mW/cm^2^ at 700 °C), Sr_2_Fe_1.5_Mo_0.5_O_6–δ_ (835 mW/cm^2^ at 900 °C), SrFe_0.9_Nb_0.1_O_3−δ_ (407 mW/cm^2^ at 800 °C), Sm_0.5_Sr_0.5_Fe_0.8_Cu_0.2_O_3−δ_ (808 mW/cm^2^ at 700 °C) and SrNb_0.1_Fe_0.9_O_3−δ_ (919 mW/cm^2^ at 600 °C) [8,26,27,28,29,30,31,32]. We ascribe the excellent electrocatalytic activities of the directly assembled LSF electrode mainly to the enlarged porosity and high specific surface area, leading to more active sites.

The electrochemical impedance spectra under open circuit conditions as a function of temperature are shown in Figure 3b. The ohmic resistance R_Ω_ is 0.128, 0.176 and 0.270 Ω·cm^2^ at 800, 750 and 700 °C, respectively. The polarization impedance R_p_ is 0.133, 0.177, 0.27 Ω·cm^2^ at the corresponding temperature. The calculated activation energy for the total polarization process of the directly assembled LSF cell is 0.726 eV (Figure 3c). To elucidate the contributions of elementary electrode processes, transformation to the distribution of relaxation times (DRT) analysis was applied. In Figure 3d, six peaks labelled as P_1_ to P_6_ can be identified clearly for each curve, and their peak areas reflect the relative polarization loss but cannot be quantified [33]. The impedance contributions have been discussed previously for a full solid oxide cell with six DRT signal peaks [34]. The high-frequency processes P_1_ (~16 kHz) and P_2_ (~1.8 kHz) are related to the oxygen ion transportation through the electrolyte or across the interfaces, while P_3_ (~600 Hz) is associated with the charge transferring in the electrodes. P_4_ (~15 Hz) relates to the diffusion and exchange of oxygen species at the three-phase boundaries (TPBs). The low-frequency processes P_5_ (0.1–1 Hz) and P_6_ (0.01–0.1 Hz) can be ascribed to the mass transfer of the reactants and gas conversion in the electrode. Obviously, as the temperature is elevated, significant decrease can be seen for process of P_1_, P_2_ and P_3_. However, the peak areas of the low-frequency processes (P_4_, P_5_, P_6_), dependent on the flux of the reactants, are not notably affected by the change of temperature. It is interesting that the summit frequencies of P_5_ and P_6_ decrease with increasing temperature, which can be ascribed to the enhanced mobility of gas molecules [33].

#### 3.2.2. SOEC Performance

**Figure 4:**
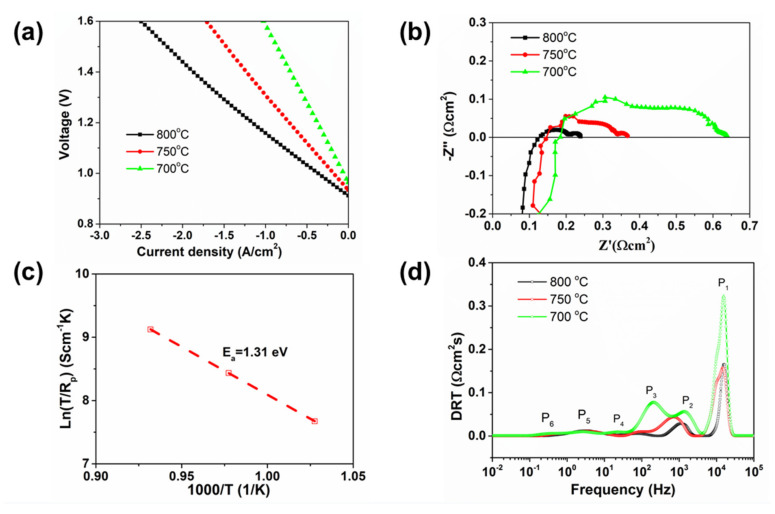
(**a**) I–V charge curves, (**b**) the impedance spectra, (**c**) the activation energy for the polarization process and (**d**) the DRT analysis of the impedance spectra for CO_2_ electrolysis at 700–800 °C.

Figure 4a presents the temperature-dependent electrochemical performance of the button cell for CO_2_ electrolysis by feeding a mixture of H_2_ and CO_2_ to the hydrogen electrode. The electrolysis current densities are 1.52, 0.98 and 0.53 A/cm^2^ at 800, 750, 700 °C and 1.3 V. The performance is also as good as that of many other cobalt-free electrodes reported previously, such as infiltrated La_0.6_Sr_0.4_FeO_3−δ_ (0.66 A/cm^2^ at 800 °C), La_0.5_Sr_0.5_Fe_0.8_Cu_0.15_Nb_0.05_O_3−δ_ (0.85 A/cm^2^ at 800 °C), Sr_2_Fe_1.5_Mo_0.5_O_6–δ_ (0.88 A/cm^2^ at 900 °C), La_2_NiO_4+δ_ (0.39 A/cm^2^ at 800 °C), Nd_2_NiO_4+δ_ (0.64 A/cm^2^ at 800 °C), La_1.2_Sr_0.8_NiO_4_ (1.37 A/cm^2^ at 700 °C) and Pr_1.2_Sr_0.8_NiO_4_ (1.12 A/cm^2^ at 700 °C) [8,35,36,37,38]. The EIS results are presented in Figure 4b. The ohmic resistance R_Ω_ is 0.131, 0.145 and 0.185 Ω·cm^2^ at 800, 750 and 700 °C, respectively, while the polarization impedance R_p_ is 0.117, 0.222, 0.453 Ω·cm^2^ The calculated activation energy for the electrolysis process is 1.31 eV, higher than that in the fuel cell mode. In our opinion, after testing in SOFC mode, the sintering of the LSF electrode will affect the microstructure and increase the polarization impedance of the cell. Another possible reason for the higher activation energy could be related to the CO_2_ electrolysis reaction, for which the cell area-specific resistance value is even bigger than that of steam electrolysis [39]. The DRT analysis is also performed and shown in Figure 4d, proving that temperature has a great influence on the ion transportation and charge transfer resistance. For the directly assembled LSF cell, the insufficient oxygen ion conductivity of pure LSF might be one factor that results in the resistance. Therefore, the composite electrode, such as LSF-GDC, will yield lowered resistance. Another strategy is to develop novel mixed ionic–electronic conducting (MIEC) materials for intermediate temperature solid oxide cells.

### 3.3. Microstructure Analysis

**Figure 5:**
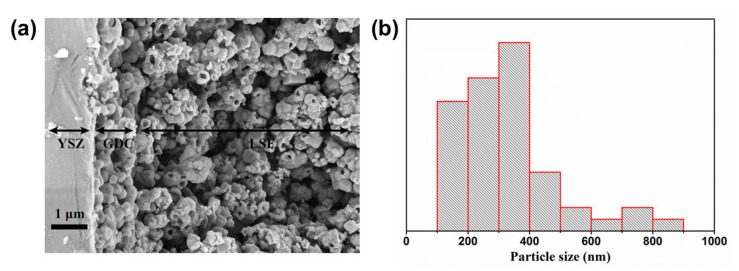
Interface of yttria-stabilized zirconia (YSZ) electrolyte/ Gd_0.1_Ce_0.9_O_1.95_ (GDC) interlayer/LSF oxygen electrode in the directly assembled button cell (**a**) and the particle size distribution of LSF grains (**b**).

Figure 5 shows the SEM image of the tested button cell with directly assembled LSF electrode after the anodic current polarization and electrochemical performance testing. We can see that the interface between the GDC interlayer and LSF oxygen electrode has been well induced in Figure 5a. Therefore, it provides evidence that the direct assembly method promises to decrease the contact resistance. In addition, most of the pores on LSF grains synthesized by the molten salt method have been well preserved, ensuring high electrochemical activities. However, the stability test for the porous particles needs to be carried out in the future. Additionally, according to the particle size distribution of LSF shown in Figure 5b, the size of most LSF particles is less than 400 nm, almost the same as the as-prepared powders. Thus, this indicates that the direct assembly method is also suitable for other nanoparticles or the materials easily decomposed at higher temperature to be used in electrodes of RSOC.

## 4. Discussion

Conventional hydrothermal and sol–gel methods can synthesize ultrafine nano powders, but it is difficult to realize mass production as easily as with the solid-state method. In comparison, the molten salt synthesis method, sharing many advantages of both the liquid-phase method and solid-state method, was utilized to produce nanometric LSF materials with special porous morphology (Figure 2b,c). As is reported, both the existence of iron and the molten salt approach play important roles in producing porous structures [10]. In a traditional RSOC oxygen electrode preparation process, high-temperature sintering limits the use of nanostructured materials. Therefore, the current polarization process, as effective as high-temperature presintering on the formation of the interface of electrode and electrolyte, was used to maintain the microstructure of the LSF electrode (Figure 5a), leading to the excellent performance of the button cell.

The key factor of the direct assembly method can be attributed to the Joule heat caused by the polarization current applied to the screen-printed electrode. Since the resistance of the electrolyte and the interface of the LSF electrode and GDC interlayer are larger than those of the electrodes, more Joule heat will be generated on the electrolyte and the interface. The phenomenon is consistent with resistance welding technology, which is commonly used in industry [40]. In practical application of the resistance welding technique, it is generally necessary to apply pressure to fix the components, and then the heat generated by the current passing through the contact of the weldment will be used for local heating welding. As for the directly assembled RSOC electrode, it has been fixed onto the electrolyte using a binder in the electrode ink. Thus, the welding effect on the interface of LSF electrode and GDC interlayer is mainly related to the applied polarization current and the contact resistance for the interface. Additionally, the contact of directly assembled electrodes might be improved owing to the high current polarization during the SOFC measurement. Since resistance welding technology has many advantages, such as being free of welding flux, high productivity, smaller deformation of weldment and ease of automation, we deduce that the direct assembly technology is also very promising in the fabrication of solid oxide cells.

In general, the combination of the MSS method and the direct assembly technique is proposed to provide solutions to the utilization of nanosized materials as electrode of solid oxide cells. In future, we will carry out detailed research on the relationship among the temperature, the polarization current, the interface contact resistance and the performance of direct assembly electrodes.

## 5. Conclusions

Porous LSF powders are prepared using a novel molten salt synthesis method as electrode materials of a reversible solid oxide cell. The direct assembly method is proposed to manufacture the LSF oxygen electrode to protect the nanostructure of the materials. Experimental results show that the directly assembled cell with porous LSF oxygen electrode reaches a maximum output power density of 1.36 W/cm^2^ at 800 °C in SOFC mode, while the electrolysis current density of the directly assembled cell is 1.52 A/cm^2^ at 1.3 V for CO_2_ electrolysis. The electrochemical testing results indicate that the combination of porous powders via the molten salt synthesis method and the directly assembled technology shows promise for the preparation of high-performance reversible solid oxide cells. Upcoming research will focus on durability and mechanism of the novel porous powders and directly assembled electrodes.
